# Analysis of questions about use of drugs in breastfeeding to Norwegian drug information centres

**DOI:** 10.1186/s13006-017-0143-8

**Published:** 2018-01-09

**Authors:** Jan Anker Jahnsen, Sofia Frost Widnes, Jan Schjøtt

**Affiliations:** 1 Regional Medicines Information and Pharmacovigilance Centre (RELIS Vest), Haukeland University Hospital, Bergen, Norway; 2 Section of Clinical Pharmacology, Laboratory of Clinical Biochemistry, Haukeland University Hospital, Bergen, Norway; 3 Department of Clinical Science, Faculty of Medicine and Dentistry, University of Bergen, Bergen, Norway

**Keywords:** Drug information centre, Drug information, Medicines, Lactation, Safety, Doctors, General practitioner, Midwife

## Abstract

**Background:**

Health professionals may advise women to either stop breastfeeding or drug treatment due to restrictive advice in drug monographs. Regional medicines information and pharmacovigilance centres in Norway (RELIS) provide free and industry-independent answers to questions about drugs and breastfeeding documented in a full-text, searchable database (RELIS database). We used the RELIS database to describe which health care practitioners sought information about medication safety in lactation, most common drugs involved, advice provided and which resources were used to provide the advice.

**Methods:**

A random selection of 100 question-answer pairs (QAPs) from the RELIS database indexed with “BREASTFEEDING” in the period from January 2011 to December 2015 was analysed. Inclusion criteria were queries from health professionals about drugs. Questions about herbal supplements and other exposures not classified as drugs were excluded. The QAPs were manually analysed for compatibility of one or several drugs with breastfeeding, health care profession and workplace of enquirer in addition to advice and search strategy used.

**Results:**

In the 100 QAPs there were enquires about 152 drugs. Seventy-four questions concerned a single drug, but the number of drugs evaluated varied between 1 and 16. Fifty-nine questions were from physicians, 34 from nurses or midwives, two from pharmacists and two from other health professionals. Questions from physicians contained 93 drug evaluations (61%), nurses or midwives 47 (31%) and pharmacists seven (5%). The most frequent categories of drugs were antidepressants, antiepileptics and immunosuppressants. The most asked about drugs were lamotrigine, codeine, quetiapine and escitalopram. Fifty-nine percent of the drugs were deemed safe while breastfeeding, 16% if precautions were taken and 12% not recommended. Thirty-nine percent of the drug evaluations used an advanced literature search strategy, and this was significantly (*p* < 0.05) more likely when the enquirer was a physician.

**Conclusions:**

This analysis of questions to Norwegian medicines information centres about medicine use in breastfeeding indicates the need for communication about safety of drugs affecting the nervous system, primarily to medical doctors and midwives. In the majority of cases the medicine information centre can reassure about the safety of breastfeeding while taking a drug.

**Electronic supplementary material:**

The online version of this article (10.1186/s13006-017-0143-8) contains supplementary material, which is available to authorized users.

## Background

Only a minority of drugs should be avoided while breastfeeding [1–3]. However, due to fear of harming their newborn many women decide to stop breastfeeding or discontinue their own drug therapy. In addition, health professionals often give women advice to cease breastfeeding because of a restrictive attitude and biased risk perception towards use of drugs in lactation [1–3]. This could stem from a lack of knowledge among health professionals regarding this topic, not having access to good sources of information or using sources that do not provide evidence-based information [1, 2].

The Summary of Product Characteristics (SPC) and Patient information leaflets (PILs) often warn against drug use while breastfeeding [1, 4], or the information provided is ambiguous [4]. This is also true for drug information sources containing abbreviation of SPCs for quick access to information about marketed drugs, like The Norwegian Pharmaceutical Product Compendium. The compendium contains monographs for each product with a particular section concerning pregnancy and breastfeeding. Warnings in this source are likely caused by lack of clinical studies investigating the exposure of newborns to drugs through mother’s milk. Furthermore, legal concerns linked to product information and market authorization is of concern for the pharmaceutical industry. The Norwegian Pharmaceutical Product Compendium is extensively used as source of drug information by Norwegian general practitioners [5], and the warnings could raise concern among health professionals.

In addition to drug information and advertising provided by the pharmaceutical industry, there are independent sources of lactation specific drug information including medical information and pharmacovigilance centres. In Norway there is a network of four regional medicines information and pharmacovigilance centres, collectively named RELIS [6]. The centres are located at university hospitals, where pharmacists and clinical pharmacologists answer questions from health care professionals working in hospitals, hospital pharmacies, general practice, community pharmacies and public health centres [7]. About 7% of the questions received by RELIS (3460 in 2016) are specific to drug safety in lactation [8].

The staff at RELIS has extensive experience with searching scientific literature and advising health professionals about the use of drugs. When RELIS answers a question regarding use of drugs and breastfeeding a search for relevant documentation is performed, including clinical studies and case reports. RELIS then concludes, or gives advice, based on the amount and quality of documentation available. Important considerations like the relative infant dose and the pharmacokinetics of the medicine are part of the advice, as well as clinical information like indications for treatment, the age of the child and frequency of breastfeeding. Notably, questions-answer pairs (QAPs) from this service are stored in a full-text, searchable database (RELIS database) where staff can find specific information about drugs and breastfeeding produced by RELIS [7]. The service aims to give decision support based on the clinical context including individual patient factors, and uses co-signatures in evaluation of the answers [9]. Thus, the service is somewhat different than breastfeeding telephone services that provide quick, but more general answers.

RELIS’ advice to health professionals is dependent on the available evidence for a drug in breastfeeding as well as professional considerations. Since experience with use of product-independent sources concerning drugs in breastfeeding is sparse, we designed a study to get an overview of questions asked including who is asking, drugs involved, advice provided and what documentation the advice are supported by.

Importantly, the results could be used to plan drug information strategies for breastfeeding women.

## Methods

### Question-answer pairs (QAPs)

The QAPs from the RELIS database are indexed and published in Norwegian. Published QAPs are freely available to health professionals in Norway and searchable through the RELIS website [6]. For each QAP, health care profession and workplace of the enquirer are registered. The professions available for selection in the database are physician, pharmacist, dentist, nurse or other. To become a midwife in Norway a bachelor degree in nursing is required, and midwives are registered as nurses in the RELIS database. The drugs for each QAP are registered according to their generic name, trade name and the ATC-number according to the Anatomical Therapeutic Chemical (ATC) classification developed by the World Health Organization [10]. Each QAP constitutes at least one evaluation of a drug’s compatibility with breastfeeding, and these drug evaluations are the basis of the statistical analysis. All QAPs from health professionals indexed with “BREASTFEEDING” in a 5 year period from January 2011 to December 2015 constituted the study material. A randomly selected sample of 100 QAPs was collected with the use of Research Randomizer [11]. This selected sample was obtained to get more detailed description of QAPs and was compared to all QAPs in the study period. If the question in a QAP was not about evaluating compatibility of a drug with breastfeeding, even though it was indexed as such, the QAP was excluded from the study. Also questions regarding herbal supplements and other exposures not classified as drugs according to the ATC classification were excluded. The QAP was excluded if the drug therapy was discouraged for other reasons than the safety of the nursing infant. Finally, direct queries from patients were also excluded from the study. When a randomly selected QAP was excluded, it was replaced with the next QAP from the chronological list of QAPs from 2011 to 2015 available in the RELIS database. Replacement with the next QAP is based on the fact that queries to RELIS are spontaneous from health professionals.

### Analysis of the questions

All questions in the database indexed with “BREASTFEEDING” from the study period were characterized as described above. If the enquirer informed about the timing of the query, as before or after the birth of the infant, this was registered. Likewise, the age of the nursing infant was registered when the enquirer informed about this in the question.

### Analysis of the answers

The written answers contain an evaluation of each drug (in case of several) and its compatibility with breastfeeding in the presented clinical setting. In some QAPs the evaluation is presented as a stand-alone conclusion, while in others it is necessary to extract the conclusion from the body of text presented to the enquirer. This meant that the text in each answer had to be manually analysed for the conclusion of compatibility for the drug with breastfeeding. This was first performed separately by SFW and JAJ, before a joint analysis led to a consensus for each drug. A pilot of 10 QAPs was performed where a clinical pharmacologist, JS, evaluated the consensus for each QAP.

Conclusions with regard to compatibility with breastfeeding were divided in to the categories “safe”, “take precautions”, “not recommended” and “no conclusion”. The answers were also analysed for any information given about the drug being excreted into human breast milk. The types of references given in the answers define the type of information search performed in each instance. References were defined as described in a previous publication from RELIS [12]. The literature search was defined as “simple” if it only referred to previous enquiries in the RELIS database, non-peer-reviewed publications by RELIS employees, databases, SPC or product labels for the drug and tertiary sources for drugs and breastfeeding like for instance “Drugs in pregnancy and lactation” by Briggs and Freeman or “Medication and mother’s milk” by Hale and Rowe. If searches in databases like Medline and Embase to obtain original or review articles were necessary, the literature search was defined as “advanced”.

### Statistical analysis

The data were analysed using SPSS 23.0 [13]. The chi-square test was applied when comparing categorical variables. Inter-rater agreement for manually analysis of text for compatibility of a drug with breastfeeding was performed with Cohen’s kappa coefficient. *P* values <0.05 were considered significant.

## Results

### Question-answer pairs (QAPs)

In the period from January 2011 to December 2015, RELIS received a total of 911 questions regarding use of drugs or other therapeutic agents in breastfeeding. To obtain a random sample of 100 QAPs we excluded 17 QAPs based on the following exclusion criteria: nine questions were not about evaluating compatibility of a drug with breastfeeding, six questions were from patients and two questions were about exposure to supplement (lecithin) and recreational substance (cannabis), rather than drugs classified within the ATC-system.

### Analysis of the questions

In the randomly selected 100 QAPs, there were enquiries about 152 drugs, resulting in an average of 1.5 drugs per QAP. Although most enquiries were about a single drug (*n* = 74), there were also 17 questions about two drugs, 5 about three drugs, 2 about four drugs and 1 question about five drugs. Separating itself from the cluster of 1–5 drugs per question was an enquiry regarding optimum choice of drug to a breastfeeding patient, which contained an evaluation of 16 drugs. Fifty-nine questions were from physicians, and these questions contained in total 93 drug evaluations (61%). Thirty-four questions involving a total of 47 drugs (31%) came from nurses or midwives, five questions involving a total of seven drugs came from pharmacists and two questions containing a total of five drugs came from other health professionals. In the majority of drug evaluations the age of the child was not known, comprising 69 of the 152 drug evaluations (45%). Eight (5%) drug evaluations concerned nursing premature infants, 25 (16%) concerned full term to 2 months old children, 35 (23%) were two to 6 months old children and 16 (10%) were children older than 6 months.

In Tables 1, 2 and 3, data are shown both for the 152 drug evaluations of the random study selection and for all queries 2011–15. The percentages of all queries 2011–15 are calculated from the number of ATC-classified drugs at the 1st, 3rd and 5th level in all QAPs indexed with breastfeeding (*n* = 911) in the RELIS database in that time period. Notice that for all queries 2011–15 the hierarchy for ATC-classification [10] means that e.g. several antidepressant drugs (e.g citalopram, mirtazapine) evaluated in one QAP from the RELIS database will lead to a single registration at the 1st and 3rd ATC level.

**Table 1:** Drugs most frequently evaluated in relation to breastfeeding at the level of the anatomical main group

	ATC level 1st Anatomical main group n (%)			
Rank	Random study selection (*n* = 152)		All queries 2011–2015 (*n* = 980)	
1	Nervous system	47 (30.9)	Nervous system	361 (36.8)
2	Cardiovascular system	30 (19.7)	Anti-infectives for systemic use	95 (9.7)
3	Anti-infectives for systemic use	17 (11.2)	Respiratory system	89 (9.1)

*ATC* Anatomical Therapeutic Classification

**Table 2:** Drugs most frequently evaluated in relation to breastfeeding at the level of the pharmacological subgroup

	ATC level 3rd Pharmacological subgroup n (%)			
Rank	Random study selection (n = 152)		All queries 2011–2015 (*n* = 1124)	
1	Antidepressants	11 (7.2)	Antidepressants	133 (11.8)
2	Antiepileptics	10 (6.6)	Antihistamines for systemic use	68 (6.0)
3	Immunosuppressants	9 (5.9)	Antiepileptics Antipsychotics	62 (5.5)

*ATC* Anatomical Therapeutic Classification

**Table 3:** Drugs most frequently evaluated in relation to breastfeeding according to the chemical substance

	ATC level 5th Chemical substance n (%)			
Rank	Random study selection (n = 152)		All queries 2011–2015 (*n* = 1340)	
1	Lamotrigine	6 (3.9)	Escitalopram	42 (3.1)
2	Codeine + paracetamol Quetiapine Escitalopram	5 (3.3)	Sertraline	36 (2.7)
3	Nifedipine Azathioprine	4 (2.6)	Lamotrigine	31 (2.3)

*ATC* Anatomical Therapeutic Classification

Table 1 shows the most frequently evaluated drugs in our random selection and among all queries in the study period indexed with breastfeeding according to the anatomical main group (1st level) in the ATC classification system. Categorizing the drugs according to the anatomical main group shows that 47 of the 152 drugs (30.9%) act on the nervous system. Drugs acting on the cardiovascular system are second with 30 drug evaluations, although as previously mentioned 16 of those were contained in a single QAP. Drugs acting on the nervous system were also the most prevalent among all the queries 2011–15, with 361 drug evaluations (36.8%).

Table 2 shows the most frequently evaluated drugs in our random selection and all queries in the study period indexed with breastfeeding, according to the pharmacological subgroup (3rd level) in the ATC classification system. Antidepressants are the most prevalent pharmacological subgroup, both in our study selection and among all queries 2011–15.

Table 3 shows the most frequently evaluated drugs in our random selection and all queries in the study period indexed with breastfeeding according to the chemical substance (5th level) in the ATC classification system. Lamotrigine is the most prevalent drug in our study selection with six drug evaluations (3.9%), and the third most prevalent among all queries 2011–15 with 42 drug evaluations (2.3%). Escitalopram, quetiapine and the analgesic combination of codeine and paracetamol each have five drug evaluations (3.3%) in our study selection. Escitalopram and sertraline are the two most prevalent drugs among all the queries 2011–15, with 42 (3.1%) and 36 (2.7%) drug evaluations respectively.

### Analysis of the answers

In 59% of the evaluations RELIS concluded that the drug in question was safe to use while breastfeeding, in 16% it was considered safe if certain precautions were implemented, in 12.5% it was not recommended and in 12.5% there was no conclusion with regard to safety. Inter-rater agreement for manual analysis of the text for compatibility of a drug with breastfeeding was 0.74. In 99 of the evaluations (65%) the ability of the drug in question to transfer to breast milk was mentioned in the answer.

As shown in Table 4, in 15.8% of the evaluations the only citations were previous answers from the RELIS database or non-peer reviewed articles published by RELIS staff, while 42.8% cited monographs and databases. Thus, 58.6% of the evaluations were based on a simple search strategy, while 39.5% of the evaluations were based on an advanced search strategy.

**Table 4:** Type of search and citations in drug evaluations concerning breastfeeding (n = 152)

	Type of search n (%)			
Search	No search	Simple		Advanced
	3 (2.0)	89 (58.6)		60 (39.5)
Citation	No citation	Previous answers from the RELIS database or non-peer reviewed articles by RELIS staff	Monographs and databases	Original articles or peer reviewed review articles
	3 (2.0)	24 (15.8)	65 (42.8)	60 (39.5)

In Table 5, the data has been grouped in order to perform a statistical analysis. The health profession of the enquirer is grouped into either “physician” or “other health professional”. The type of search is divided into “simple” or “advanced”, where “advanced” is the same category as presented in Table 4, and “simple” in addition includes the category “no citation”. As can be seen in Table 5, when a physician is the enquirer it is more likely that a peer reviewed original or review article is cited in the answer (“advanced search”), when compared to other health professionals (chi-square test, *p* = 0.032).

**Table 5:** Type of search and health profession of enquirer in drug evaluations concerning breastfeeding (n = 152)

Health profession n (%)	Type of search n (%)	
	Simple^b^ 92 (60.5)	Advanced^c^ 60 (39.5)
Physician 93 (61.2)	50 (53.8)	43 (46.2)*
Other health professionals^a^59 (38.8)	42 (71.2)	17 (28.8)

^a^Nurses or midwives, pharmacists and health professionals not further identified in the database

^b^Simple search strategy includes no citations, RELIS database, non-peer reviewed articles, monographs and databases

^c^Advanced search strategy includes peer reviewed original or review articles

*Chi-square test, p = 0.032 for difference between physicians and other health professionals with regard to type of search

The data was also analysed for correlation between compatibility with breastfeeding and the health profession of the enquirer, as well as between compatibility with breastfeeding and the search strategy. The profession (physician or not) of the enquirer did not influence on the conclusion regarding compatibility of a drug with breastfeeding (chi-square test, *p* = 0.752). A simple search strategy was more likely to be performed when the drug was deemed compatible with breastfeeding (chi-square test, *p* ≤ 0.001). Conversely, an advanced search strategy was more likely to be performed when the drug was considered problematic, or not recommended, to be used while breastfeeding.

## Discussion

The main findings in the present study were that about 60% percent of questions about use of drugs in breastfeeding came from physicians and about 30% from nurses or midwives. Health professionals ask on average about 1.5 drugs per question, and they most frequently ask about drugs with effects in the nervous system. About 60% of drug evaluations conclude that drugs are compatible with breastfeeding, and only about 10% are contraindicated. Questions from physicians are associated with a more extensive literature search strategy compared to other health professionals.

To the best of our knowledge this is one of the first analyses of written QAPs to a drug information center from health professionals regarding use of drugs in breastfeeding. Several descriptive studies of telephone services providing drug or teratology information to the public and/or health care professionals have been published [14–19]. These publications provide statistics and analysis of enquiries from the public and/or health care professionals, but are based on telephone services giving oral advice to the caller. RELIS has chosen a different strategy than the traditional telephone based drug or teratology information services [7]. RELIS is a service solely for health professionals and we receive 68% of the questions through a web question form at the website or e-mail [8]. Advice to the enquirer is provided as written answers with co-signatures who check text structure, content and reference use. The different profiles of the services with regard to handling of questions and documentation of answers make a comparison of the studies difficult.

As can be seen in Table 1, drugs affecting the nervous system and anti-infectives for systemic use are most prevalent in our study selection and in all queries in the study period. The high number of queries about drugs affecting the cardiovascular system in the random selection can be traced to a single query containing evaluations of 16 different cardiovascular drugs. A general practitioner had a hypertensive patient, who gave birth 3 months prior to the query. The physician asked RELIS for advice when choosing a medication because most antihypertensive drugs are contraindicated to nursing women in product monographs. As the query did not indicate any preferred drug, the answer contained evaluations of drugs from all classes of antihypertensives.

In our experience, the queries to RELIS about compatibility of drugs with breastfeeding concerns drugs with uncertain safety assessment in the drug monographs. The drugs that actually are prescribed most frequently to mothers the first 3 months postpartum in Norway are seldom in question. These are drugs affecting the genitourinary system and sex hormones, followed by anti-infectives for systemic use and systemic hormonal preparations, excluding sex hormones, and insulins [20]. Drugs affecting the respiratory system, cardiovascular system and nervous system are only the fifth, sixth and seventh most prescribed drugs. The most frequently prescribed drugs after birth can collectively be termed pregnancy-related drugs [20]. Established routines and experience in both hospitals and among general practitioners and midwives concerning this prescribing would reduce the need for information about these drugs. Anti-infectives for systemic use are among the most prescribed and most asked about, which could be a reflection of the diversity of chemical substances and therapeutic indications among this group of drugs.

The three most queried drugs at the pharmacological subgroup level in our study selection and among all queries in the study period were antidepressants and antiepileptics (Table 2). Immunosuppressants are third in our random selection, but for the entire period it is the seventh most queried (Additional file 1). The high number of queries for immunosuppressants is surprising, since these drugs are among the least prescribed in the first 3 months postpartum [20].

At the chemical substance level, both in our study selection and all queries in the study period, questions are dominated by drugs affecting the nervous system, with the only exceptions being nifedipine and azathioprine as the third most prevalent in our study selection (Table 3). The most frequently asked about drug is lamotrigine, which is frequently used in epilepsy and bipolar disorder [21]. Lamotrigine is one of the drugs recommended in Norwegian guidelines for treating pregnant women with bipolar disorder [22]. The guidelines also state that breastfeeding while taking lamotrigine is usually not recommended [22]. In 2009 a case report which involved RELIS was published about severe apnea in a 16-day-old infant exposed to lamotrigine in breast milk [23]. As the case originated from a Norwegian hospital and was presented by RELIS, it is possible that this case received more attention among Norwegian health professionals than the average case report would. Additionally, treatment with lamotrigine during pregnancy requires increasing the dosage due to increased clearance of the drug. To avoid adverse effects in the mother and higher than acceptable levels of lamotrigine in the breast milk, it is important to quickly reduce the dose after the birth.

The two most commonly questioned drugs among all the queries in the study period are the SSRIs escitalopram and sertraline, and escitalopram is also the second most frequent in our study selection. Psychiatric illness (depression or psychosis) during pregnancy and in the postnatal period is not uncommon. A recent meta-analysis found an incidence of about 16% among patients with known risk factors and about 3% among patients without known risk factors [24]. Today there is a consensus that women with a need for an SSRI can continue treatment in the pregnancy, and the focus has shifted from potential risk of the pharmacological treatment to the risk of untreated psychiatric illness [25–27]. One explanation for a high prevalence of questions is that this focus is not reflected in the sections concerning breastfeeding in the respective drug monographs.

Quetiapine is the second most asked about drug in relation to breastfeeding in our study material. In recent years quetiapine has been widely used off-label in low dosage for sleep and sedation [28, 29]. Insomnia is prevalent among breastfeeding women [30], and is associated with psychiatric diseases and affects quality of life [31]. A study found a prevalence of insomnia at around 60% 8 weeks postpartum [32]. It seems likely that off-label use of quetiapine can explain the high number of questions regarding this drug. Among all the queries about breastfeeding in the five-year period, quetiapine is the fourth most asked about drug (Additional file 1). This corresponds well with the increase in off-label low dosage prescribing of quetiapine in Norway [33]. From 2004 to 2015 there was more than a 10-fold increase in users of quetiapine, and in the same time period the dispensed defined daily dose (DDD) per day was reduced by two thirds [33]. In both the Norwegian study and an earlier British study [34] the majority of quetiapine users had no major psychiatric diagnosis. Quetiapine off-label use has been advocated for a number of conditions, including anxiety disorders, depression and insomnia, and in 2010 AstraZeneca was fined USD $520 million for illegal marketing of quetiapine for non-approved conditions [33].

Preparations of codeine and paracetamol are the established treatment in Norway when non-opioid pain treatment is insufficient. In 2007, a case report described a breastfed neonate who died of morphine overdose due to the mother’s use of codeine [35]. This emphasized the risks involved with using codeine in breastfeeding women, in particular because codeine is partly metabolized to morphine with potential sedative and respiratory depressive effects on the infant. This metabolic pathway is subject to pharmacogenetic polymorphism, and the mother in the aforementioned case was an ultrarapid metabolizer of codeine, resulting in higher than normal morphine values in plasma and breast milk [35].

In Norway the recommended antihypertensive treatment for pregnant and postpartum women is nifedipine, labetalol or methyldopa [36]. Nifedipine and labetalol are considered equally effective in treating severe or acute hypertension in pregnant or postpartum women [37], and the Norwegian guidelines recommend the prescriber to choose the drug the physician is most familiar with [36]. In addition to this, nifedipine is the pharmacological treatment of choice for Raynaud’s phenomenon, and Raynaud’s phenomenon also occurs in the nipple in breastfeeding mothers [38–40]. A different name for this painful condition is vasospasm of the nipple, and an Australian study found a prevalence of 20% 8 weeks postpartum among first-time mothers [41]. Nifedipine is the most asked about drug affecting the cardiovascular system, both in our random selection and among all the queries 2011–15 (Additional file 1).

Even though azathioprine is considered safe for use in both pregnant and breastfeeding patients, there seems to be uncertainty among health professionals about its use in these patients. A Canadian study investigating physicians’ management of inflammatory bowel disease during pregnancy and breastfeeding concluded there was a need for targeted educational activities towards general practitioners, while gastroenterologists demonstrated a high level of knowledge [42]. While about 90% of the gastroenterologists would continue treatment with azathioprine during pregnancy, only about 16% of the general practitioners would [42].

When physicians ask RELIS questions whether medicines are compatible with breastfeeding, we use a more advanced type of literature search compared to when other health professionals ask us. We have not distinguished between general practitioners and specialists in our material, so we do not know how the results would have been with further differentiation. It seems plausible that physicians with the responsibility of treating a nursing mother ask more detailed questions and expect a more detailed answer, than health professionals (e.g. midwives, community pharmacists) who are often consulted by women with common postpartum conditions. Furthermore, other health professionals than physicians are more likely to be consulted about symptomatic treatment (e.g. headache, hay-fever) compared to more complex questions about treatment of chronic diseases in breastfeeding women. Our experience is that questions about symptomatic treatment of common self-limiting diseases are well described in drug information sources with regard to breastfeeding. Thus, a simple search is often sufficient with regard to this type of questions.

The material in this study is a randomly selected sample of 100 QAPs from the RELIS database, about 11% of the QAPs about breastfeeding in the period 2011–2015. Notably, questions to RELIS are spontaneous and may not be representative of the typical care provider in their respective professions. Furthermore, in the case of a drug being discussed in media or a sudden change in national drug monographs or marketing in Norway we could expect frequent questions about that drug. Our randomization of questions reduces the impact of this bias in our material. However, since a health professional can ask about choice of a drug within a category (e.g. antihypertensives), several drug evaluations from a single QAP can influence a selection. A further weakness in our method is the need for text analysis of the answer given for each query. The text must be analysed and the conclusion must be extracted from the body of text formulated by the individual staff member in RELIS. However, we found a high degree of inter-rater agreement in the text analysis. To reduce any uncertainties we first performed this analysis separately before a joint analysis led to a consensus for each drug, corroborated by a statistical analysis of the inter-rater agreement. In a number of cases the text was too ambiguous for us to extract a conclusion, which resulted in 13% of the drug evaluations being categorized as having no conclusion.

Although 65% of the drug evaluations in this study mentioned the ability of the drug to transfer to breast milk, it was not possible to discern between mere mentioning this information from it being a deciding factor. In Norway there is a well-established and strong culture of breastfeeding, and a general practitioner is expected to adapt any treatment to accommodate lactation as much as possible. This focus on breastfeeding despite chronic or acute illness could influence the prevalence of questions and type of drugs enquired about, compared to countries with less focus on breastfeeding. Finally, more information about the outcome of our advice in terms of health consequences would be of interest.

## Conclusions

This analysis of questions posed to Norwegian medicines information centres about use of drugs in breastfeeding describes a need for improving knowledge and communication regarding the safety of medications in lactation to practitioners (e.g. physicians and midwives) who care for postpartum women. Although the complexity of the questions and the availability of documentation often require an advanced literature search, in particular with questions from physicians, the medicines information centre in the majority of cases was able to reassure the practitioner with drug safety information in breastfeeding. Based on the fact that the service is free of charge and offers less categorical advice than product monographs from the drug industry this could represent a useful and important drug information source that contributes to the health of both breastfeeding women and their infants.

## Additional file

Additional file 1: RELIS all queried drugs breastfeeding 2011–15 (XLSX 14 kb)

## Abbreviations

ATC: Anatomical therapeutic chemical
DDD: Defined daily dose
PIL: Patient information leaflet
QAP: Question-answer pair
RELIS: Regional medicines information and pharmacovigilance centres
SPC: Summary of product characteristics
SSRI: Selective serotonergic reuptake inhibitors

## References

[1] Davanzo R, Bua J, De Cunto A, Farina ML, De Ponti F, Clavenna A (2016). Advising mothers on the use of medications during breastfeeding: a need for a positive attitude. J Hum Lact.

[2] Hussainy S, Dermele N (2011). Knowledge, attitude and practices of health professionals and women towards medication use in breastfeeding: a review. Int Breastfeed J.

[3] Della-Giustina K, Chow G (2003). Medications in pregnancy and lactation. Emerg Med Clin N Am.

[4] Arguello B, Salgado TM, Fernandez-Llimos F (2015). Assessing the information in the summaries of product characteristics for the use of medicines in pregnancy and lactation. Br J Clin Pharmacol.

[5] Høye S, Straand J, Brekke M (2008). How do general practitioners keep up-to-date on pharmacotherapy?. Tidsskr Nor Legeforen.

[6] Regionale legemiddelinformasjonssentre (RELIS). www.relis.no. Accessed 12 July 2016.

[7] Schjøtt J, Reppe LA, Roland PD, Westergren T (2012). A question-answer pair (QAP) database integrated with websites to answer complex questions submitted to the regional medicines information and Pharmacovigilance Centres in Norway (RELIS): a descriptive study. BMJ Open.

[8] RELIS. [RELIS Annual report 2016.] https://relis.no/multimedia/209/RELIS_Arsrapport_2016 [Report in Norwegian]. Accessed 12 July 2016.

[9] Reppe LA, Spigset O, Schjøtt J (2016). Drug information services today: current role and future perspectives in rational drug therapy. Clin Ther.

[10] WHO Collaborating Centre for Drug Statistics Methodology. ATC/DDD Index 2016. https://www.whocc.no/atc_ddd_index/ Accessed July 2017.

[11] Research Randomizer. http://www.randomizer.org/ Accessed Jan 2017.

[12] Reppe LA, Spigset O, Schjøtt J (2010). Which factors predict the time spent answering queries to a drug information centre?. Pharm World Sci.

[13] IBM Corp. Released (2015). IBM SPSS statistics for windows, version 23.0.

[14] Rutter PM, Jones W (2012). Enquiry analysis and user opinion of the drugs in Breastmilk helpline: a prospective study. Int Breastfeed J.

[15] Campbell SC, Kast TT, Kamyar M, Robertson J, Sherwin CM (2016). Calls to a teratogen information service regarding potential exposures in pregnancy and breastfeeding. BMC Pharmacol Toxicol.

[16] Patil AS, Patil NP, Lewis AN, Swamy GK, Murtha AP (2014). Health care providers’ use of a drug information service for pregnancy-related inquiries. J Am Pharm Assoc (2003).

[17] Tan MP, Koren G (2011). Teratogen information service for pharmacists: a pilot study. J Popul Ther Clin Pharmacol.

[18] Gendron MP, Martin B, Oraichi D, Bérard A (2009). Health care providers' requests to teratogen information services on medication use during pregnancy and lactation. Eur J Clin Pharmacol.

[19] Addis A, Impicciatore P, Miglio D, Colombo F, Bonati M, Da Silva CD (1995). Drug use in pregnancy and lactation: the work of a regional drug information center (letter). Ann Pharmacother.

[20] Engeland A, Bramness JG, Daltveit AK, Rønning M, Skurtveit S, Furu K (2008). Prescription drug use among fathers and mothers before and during pregnancy. A population-based cohort study of 106,000 pregnancies in Norway 2004-2006. Br J Clin Pharmacol.

[21] Vajda FJ, Dodd S, Horgan D (2013). Lamotrigine in epilepsy, pregnancy and psychiatry--a drug for all seasons?. J Clin Neurosci.

[22] The Norwegian Directorate of Health. [Nasjonal fagleg retningslinje for utgreiing og behandling av bipolare lidingar/Norwegian guidelines for diagnosis and treatment of bipolar disorders.] http://www.helsebiblioteket.no/retningslinjer/bipolare-lidingar/forord [Guidelines in Norwegian]. Accessed July 2017.

[23] Nordmo E, Aronsen L, Wasland K, Småbrekke L, Vorren S (2009). Severe apnea in an infant exposed to lamotrigine in breast milk. Ann Pharmacother.

[24] Grekin R, O'Hara MW (2014). Prevalence and risk factors of postpartum posttraumatic stress disorder: a meta-analysis. Clin Psychol Rev.

[25] Yonkers KA, Wisner KL, Stewart DE, Oberlander TF, Dell DL, Stotland N (2009). The management of depression during pregnancy: a report from the American Psychiatric Association and the American College of Obstetricians and Gynecologists. Obstet Gynecol.

[26] The National Institute for Health and Care Excellence (NICE). Antenatal and postnatal mental health: clinical management and service guidance (Published: December 2014). https://www.nice.org.uk/guidance/cg192 Accessed July 2017. 31841290

[27] McAllister-Williams RH, Baldwin DS, Cantwell R, Easter A, Gilvarry E, Glover V (2017). British Association for Psychopharmacology consensus guidance on the use of psychotropic medication preconception, in pregnancy and postpartum 2017. J Psychopharmacol.

[28] Anderson SL, Vande Griend JP (2014). Quetiapine for insomnia: a review of the literature. Am J Health Syst Pharm.

[29] Hermes ED, Sernyak M, Rosenheck R (2013). Use of second-generation antipsychotic agents for sleep and sedation: a provider survey. Sleep.

[30] Dørheim SK, Bondevik GT, Eberhard-Gran M, Bjorvatn B (2009). Sleep and depression in postpartum women: a population-based study. Sleep.

[31] Ellis JG, Perlis ML, Bastien CH, Gardani M, Espie CA (2014). The natural history of insomnia: acute insomnia and first-onset depression. Sleep.

[32] Sivertsen B, Hysing M, Dørheim SK, Eberhard-Gran M (2015). Trajectories of maternal sleep problems before and after childbirth: a longitudinal population-based study. BMC Pregnancy Childbirth.

[33] Gjerden P, Bramness JG, Tvete IF, Slørdal L (2017). The antipsychotic agent quetiapine is increasingly not used as such: dispensed prescriptions in Norway 2004-2015. Eur J Clin Pharmacol.

[34] Marston L, Nazareth I, Petersen I, Walters K, Osborn DP (2014). Prescribing of antipsychotics in UK primary care: a cohort study. BMJ Open.

[35] Madadi P, Koren G, Cairns J, Chitayat D, Gaedigk A, Leeder JS (2007). Safety of codeine during breastfeeding: fatal morphine poisoning in the breastfed neonate of a mother prescribed codeine. Can Fam Physician.

[36] Norwegian Gynaecological Association. Guidelines for obstetric aid 2014, chapter 28 (hypertensive pregnancy complications and eclampsia). Available from: http://legeforeningen.no/Fagmed/Norsk-gynekologisk-forening/Veiledere/Veileder-i-fodselshjelp-2014/ [Guidelines in Norwegian].

[37] Firoz T, Magee LA, MacDonell K, Payne BA, Gordon R, Vidler M (2014). Oral antihypertensive therapy for severe hypertension in pregnancy and postpartum: a systematic review. BJOG.

[38] Wigley FM, Flavahan NA (2016). Raynaud’s phenomenon. N Engl J Med.

[39] Barrett ME, Heller MM, Stone HF, Murase JE (2013). Raynaud phenomenon of the nipple in breastfeeding mothers: an underdiagnosed cause of nipple pain. JAMA Dermatol.

[40] Wu M, Chason R, Wong M (2012). Raynaud's phenomenon of the nipple. Obstet Gynecol.

[41] Buck ML, Amir LH, Cullinane M, Donath SM, CASTLE Study Team (2014). Nipple pain, damage, and vasospasm in the first 8 weeks postpartum. Breastfeed Med.

[42] Huang VW, Chang HJ, Kroeker KI, Goodman KJ, Hegadoren KM, Dieleman LA, et al. Management of inflammatory bowel disease during pregnancy and breastfeeding varies widely: a need for further education. Can J Gastroenterol Hepatol. 2016;6193275 10.1155/2016/6193275 PMC5048030 27725926

